# The Potential Role of Gender in the Incidence, Management, and Outcomes of Stroke in Patients Suffering From COVID-19: A Brief Review

**DOI:** 10.7759/cureus.50302

**Published:** 2023-12-11

**Authors:** Meropi Mpouzika, Christos Rossis, Georgios Tsiaousis, Maria Karanikola, Maria Chatzi, Stelios Parissopoulos, Elizabeth Papathanassoglou

**Affiliations:** 1 Department of Nursing, Cyprus University of Technology, Limassol, CYP; 2 Department of Accident & Emergency, Nicosia General Hospital Cyprus, Nicosia, CYP; 3 Infectious Diseases Unit, University Hospital of Larissa, Larissa, GRC; 4 Department of Nursing, University of West Attica, Athens, GRC; 5 Department of Critical Care, Faculty of Nursing, University of Alberta, Edmonton, CAN; 6 Neurosciences, Rehabilitation & Vision Strategic Clinical Network, Alberta Health Services, Edmonton, CAN

**Keywords:** gender differences, sex differences, stroke, outcomes, management, literature review, incidence, critical care, covid-19

## Abstract

Gender-disaggregated data are continuously needed in all aspects of the coronavirus disease 2019 (COVID-19) pandemic, including cerebrovascular disease in patients infected with SARS-CoV-2. This brief review was conducted to summarize available evidence and highlight potential sex differences regarding the incidence, applied therapies, and outcomes of stroke in patients with COVID-19. Local and global registries of such patients were included, where comparisons with historical (pre-pandemic era) and contemporary (stroke patients negative for SARS-CoV-2) cohorts formed the basis of the analysis. According to the herein reported evidence, the frequency of stroke under COVID-19 does not seem to vary according to gender, although a tendency toward male predominance cannot be excluded. In terms of management and outcomes, more advanced therapies are used in men. Follow-up data on gender differences are needed, as the pandemic is evolving (no lockdowns; new strains; vaccinated or naturally immune populations).

## Introduction and background

Causing a predominantly respiratory disease, coronavirus disease 2019 (COVID-19), infection with severe acute respiratory syndrome coronavirus 2 (SARS-CoV-2) has spread all over the world, resulting in a pandemic, which, three years later, continues to be responsible for significantly excess morbidity and mortality and exerts stress on healthcare systems [1,2].

A neurological involvement was recognized early, and elucidation of its pathophysiological basis was achieved through intensive research, which highlighted an interplay of oxidative stress, endothelial inflammation, coagulopathy, thromboembolism, cytokine storm, and, finally, neuroinflammation [3,4]. An extensively studied neurological manifestation of COVID-19 is acute ischemic stroke [4]. Stroke in general affects women more than men: more women suffer from stroke, stroke case fatality is higher in women, and, when they survive, women recover more poorly than men [5]. The basis of this is a multitude of factors, including hormonal, coagulation, and immunity differences, as well as social determinants linked to access to diagnosis, treatment, and rehabilitation [5]. From the beginning of the COVID-19 pandemic, differences among men and women in disease course and outcome have attracted significant scientific attention [6,7], and a need for gender-disaggregated data on all aspects of the disease has risen [8-10]. Although gender has been included in the demographic variables typically reported in all studies of stroke in patients suffering from COVID-19 (termed “stroke/COVID” hereafter), the gender differential in stroke/COVID has not attracted considerable attention. Meta-analyses that have been published regarding stroke/COVID had not been specifically designed to tackle the gender differential; therefore, the majority of studies included in them did not have gender-disaggregated data [11-13]. Finally, knowledge of possible gender-specific differences would be of great importance to healthcare professionals within critical care settings, as most stroke patients are hospitalized in critical care wards, especially during the first hours after presentation, when intravenous thrombolysis and/or other endovascular therapies are administered. For the reasons mentioned above and to encourage further research, this review aims to emphasize a potential gender gradient among patients with stroke and COVID-19 by addressing the following three fundamental questions that require exploration:

1. Does stroke/COVID occur more frequently in women than in men?

2. Is there a gender difference in outcomes?

3. Is there a difference in the use of available therapies for stroke (mainly revascularization with intravenous thrombolysis or mechanical thrombectomy [MT]) among men and women with COVID-19?

## Review

Methods

Various databases were searched, up to April 30, 2023, for relevant articles: PubMed, Google Scholar, and Scopus. The keywords used included the following: “SARS-CoV-2” OR “COVID-19” AND “stroke” OR “cerebrovascular disease” OR “cerebrovascular accident” OR “cerebrovascular manifestations” AND “gender” OR “sex” OR “women” OR “men” OR “male” OR “female” AND “differential” OR “gradient” AND “hemorrhagic stroke” OR “transient ischemic attack” AND “neurological involvement” OR “neurological manifestations” AND “intravenous thrombolysis” OR “endovascular therapies” OR “thrombectomy” OR “mechanical thrombectomy.” We used predefined inclusion and exclusion criteria to identify and select studies. We included primary studies that focused on stroke in COVID-19 patients and provided relevant data on the outcomes of interest. Exclusion criteria included studies not written in English, conference abstracts, those with insufficient data, small size of stroke/COVID patient populations (<50), short (<one month) enrollment duration, studies with no proven COVID-19 cases, and, most importantly, absence of gender-specific data. We chose to critically appraise the most impactful original articles on this issue, including studies of nationwide or global extent, where cases were retrieved from local, national, and international registries of stroke cases. We included multi-center studies, either national or international. Additionally, we incorporated two single-city multi-center studies originating from New York City [14] and Chicago, U.S. [15]. We specifically chose to include the New York study [14] because it involved patients from 11 different hospitals in the New York metropolitan area, and, secondly, because New York was the first major American city to be heavily struck by the pandemic. The Chicago area study [15] was included because it involved cases from six comprehensive stroke centers, and, secondly, it was the only study with a specific design to detect outcome differences by gender. Single-center, low-volume studies, stemming from countries or cities not on the forefront of the pandemic, were chosen to be left out, unless they were included as part of the herein accepted meta-analyses. Additionally, through our database searches, we identified meta-analyses, which informed the discussion of available evidence. These studies are mentioned in the discussion section, providing a clear separation between the synthesized results and the original studies. As mentioned before, only a fraction of the studies included in these meta-analyses demonstrated gender-disaggregated data on stroke/COVID.

The search process was conducted by two independent investigators. Both investigators independently screened the titles and abstracts of the identified studies to select potentially relevant articles. In cases of discrepancies or disagreements between the investigators, a third investigator was consulted to reach a consensus. We managed duplicates in our search results by reviewing the records based on titles and abstracts to identify and exclude additional duplicates or irrelevant studies. From the selected studies, we extracted key data, including patient demographics, study design, stroke characteristics, and relevant outcomes, including stroke incidence rates, stroke subtypes, mortality rates, and neurological and functional outcomes in patients who developed stroke.

The quality of included studies was independently assessed by two researchers, and consensus was reached over through discussions. We employed the critical appraisal tools developed by the Joanna Briggs Institute to evaluate the rigor of the studies [16].

Results

We identified 11 studies meeting the eligibility criteria. The characteristics of the studies from which gender-disaggregated data on stroke under COVID-19 were retrieved are depicted in Table 1. Of the primary studies, five involved samples from the USA [14,15,17-19], four from Europe [20-23], and two had a global/ international reach [24,25]. All studies employed retrospective cohort designs, with the exception of one cross-sectional study [22]. Only for studies employed either historical or time-matched controls [14,17,18,25]. All identified studies exhibited a low risk of bias. Additionally, we identified three meta-analyses [11-13] highlighting relevant gender-related comparisons, which informed the discussion session.

**Table 1:** Studies from which gender-disaggregated data on stroke under COVID-19 were retrieved CI, confidence intervals; EVT, endovascular treatment; IVT, intravenous thrombolysis; MT, mechanical thrombectomy; mTICI, modified Thrombolysis in Cerebral Infarction; OR, odds ratio; RR, risk ratio; TIA, transient ischemic attack; UK, United Kingdom; USA, United States of America

Author(s)	Design/data sources	Period of recruitment	Setting	Geographical area of interest	Number of Stroke/COVID patients (number of controls)	Sex-specific frequency of occurrence	Sex-specific mortality	Other sex-specific outcomes	Comments
Harrison et al., 2021 [17]	Retrospective cohort study; comparison to historical cohort (2019)	Jan 20 to Oct 10, 2020	Search through electronic health records	90% USA, 10% non-USA	952 (952 propensity-matched historical controls)	No sex difference	60-day Mortality higher in stroke/COVID patients, lack of sex-specific data		Included stroke patients with SARS-CoV-2 positivity up to 30 days prior to stroke
Richter et al., 2022 [20]	Retrospective cross-sectional study; stroke diagnoses derived from an administrative diagnosis-related group database	January 1, 2020, to December 31, 2020; four periods: pre-first wave, first-wave, pre-second wave, second wave	All German hospitals; patients hospitalized for ischemic stroke	Germany	1,268 (210,254 total ischemic strokes in 2020)	Percentage of male was steady throughout 2020 (vs. 2019 controls) and no significant difference compared to females, but no gender-specific data from the stroke/COVID cohort only		A statistically significant and greater decline in female stroke hospitalizations during the second wave was found	
Katz et al., 2020 [14]	Retrospective case series; comparison to date-matched controls (2019)	March 14, to April 26, 2020	11 New York hospitals	USA, New York	86 (499 date-matched controls)	No sex difference	No sex difference	No sex difference in in-hospital stroke onset	
Mariet et al., 2022 [21]	Retrospective study; data from the French National Hospital Discharge database	January 1, 2020, to September 30, 2020	All French hospitals	France	1,361 (22,995 non-COVID-19 stroke patients)	Increased male representation in the stroke/COVID cohort vs. the non-COVID stroke cohort (58.4% vs. 51.4%, p<0.0001)			A statistically significant male predominance was found both in ischemic and hemorrhagic strokes, but not in TIAs
Qureshi et al., 2021 [18]	Retrospective cohort study; two comparisons: stroke/COVID vs. stroke/non-COVID and stroke/COVID vs. non-stroke COVID-19 patients	December 2019 to April 2020	54 healthcare facilities searched through deidentified COVID-19 dataset	USA	103 stroke/COVID patients (among 8,163 COVID-19 patients); 199 stroke patients (among 19,513 patients without COVID-19; 27,676 total patients in the dataset	No sex difference among all comparisons	Among COVID-19 patients with or without stroke, male sex had an RR of 1.1 for death or discharge to destination other than home	Discharge to destination other than home	Design encompassed two comparisons (see “Design”)
Shakil et al., 2022 [19]	Retrospective cohort study; American Heart Association COVID-19 Cardiovascular Disease Registry	January 29, 2020, to November 23, 2020	107 U.S. hospitals	USA	289 total strokes of which 160 were ischemic (21,073 hospitalized COVID-19 patients)	More men among ischemic stroke/COVID patients than among non-stroke controls (63.1% vs. 53.9%, p=0.025); association lost in adjusted models			Significant number of non-ischemic stroke patients (129) included, again more men than in the non-stroke population (63,6% vs. 53.9%, p-value not reported)
Gabet et al., 2021 [22]	Cross-sectional study; data from the French National Hospital Discharge database	January 1, 2020, to June 14, 2020	All French hospitals	France	800 (55,395 hospitalized stroke patients without COVID-19)	No sex difference		Men underwent mechanical thrombectomy more frequently than women (66% vs. 48%, p=0.02)	
Marto et al., 2025 [24]	Retrospective multi-center cohort study; the Global COVID-19 Registry	March 1, 2020, to June 30, 2021	105 centers; consecutive patients with acute ischemic stroke receiving IVT and/or EVT	Worldwide	853 (15,128)	Male predominance (57.9% among stroke/COVID vs. 51% among non-COVID strokes, p<0.001)		Men comprised 58% among EVT-only patients vs. 48.5% among controls, p<0.001	Only ischemic stroke patients who received IVT and/or EVT were included
Dmytriw et al., 2022 [25]	Multi-center retrospective study; comparison of stroke/COVID patients vs. non-COVID stroke patients	February 25, 2020, to December 30, 2020	Consecutive admissions of large vessel occlusion stroke patients in stroke centers	North America, Europe, Middle East	302 (395 non-COVID stroke patients), all with large vessel occlusion	Percentage of female fell from 80.5% in the non-COVID cohort to 41.1% in the stroke/COVID cohort (p<0.001)		Female sex was an independent predictor of favorable revascularization (mTICI grade 3)	
Trifan et al., 2020 [15]	Retrospective multi-center; no control cohorts	March 15 to May 15, 2020	6 stroke centers	USA, Chicago	83	53% males (no comparison to the control group)	Men higher (38% vs 18%)	Men exhibited higher modified Rankin Score upon discharge	Only study with a dedicated design to detect sex differences
Cagnazzo et al., 2021 [23]	Multi-center cohort registry (the “ET-COVID-19 Study”)	March 1, 2020, to May 5, 2020	Ischemic stroke patients with large vessel occlusion that were admitted to comprehensive stroke centers and underwent mechanical thrombectomy	France, Italy, Spain Belgium	93 stroke/COVID patients among 855 total MT patients	Male patients represented 67.7% of the stroke/COVID/MT cohort			No comparison to non-COVID stroke patients was performed

Differences in gender-specific frequency of stroke under COVID-19

To explore differences in gender-specific frequencies of stroke in patients with COVID-19, we assessed evidence from studies in which researchers compared cohorts of patients with stroke/COVID to properly matched either (i) historical cohorts, i.e., pre-pandemic stroke patients of the previous year(s) [14,17,20], and/or (ii) contemporary cohorts of patients with stroke but no COVID-19 [18,19,21,22]. Harrison et al. performed a retrospective cohort study using electronic records from 50 healthcare organizations in the USA. Contrasting 954 cases with COVID-19 and ischemic stroke against 48,363 historical controls without COVID-19, the study highlighted a substantial mortality risk escalation in ischemic stroke patients with concurrent COVID-19 and found no gender difference [17]. The investigators included cases with COVID-19 positivity up to 30 days before the incident of stroke. In an extensive American Heart Association registry encompassing 21,073 hospitalized COVID-19 patients, 0.75% experienced ischemic stroke or transient ischemic attack, while 0.61% had other types of stroke. The findings suggested a potential mechanism for COVID-19-related ischemic stroke that operates independently of age. Although the majority of stroke/COVID cases were male (63.1% among stroke/COVID patients compared to 53.9% among non-stroke COVID-19 controls), this association was reportedly no longer significant in multi-variate analysis [19]. In a study involving 54 U.S. centers, a double-comparison design was employed to investigate acute ischemic stroke in COVID-19 patients. The findings indicated that while acute ischemic stroke in COVID-19 cases was infrequent, it often coexisted with other cardiovascular risk factors, significantly influencing outcomes. The study compared 103 stroke/COVID patients with 7,606 controls having COVID-19 but no stroke and, subsequently, with 199 controls having stroke but no COVID-19. Interestingly, no significant difference in gender representation was observed across all comparisons [18]. A retrospective case series that involved 11 hospitals in the New York area and compared 86 stroke patients with COVID-19 to 499 date-matched controls from 2019 also demonstrated a non-significance of sex in the development of stroke. The study concluded that COVID-19 independently increased the risk of stroke and mortality in hospitalized patients [14]. A nationwide study in France by Gabet et al., including more than 55,000 stroke cases and approximately 800 stroke/COVID patients during the first half of 2020, highlighted a substantial increase in mortality among stroke patients with COVID-19. This emphasizes the need for further research to understand the excess mortality associated with these cases. The study found no gender difference in the occurrence of the combination of stroke and COVID-19 [22]. In a second French study of the impact of the COVID-19 pandemic on myocardial infarction and stroke hospitalizations, comparing data from 2019 to the period surrounding the 2020 nationwide study, significant male predominance was observed. Among patients with stroke/COVID, men represented 58.4%, whereas among non-COVID strokes, their proportion fell to 51.4% of cases [21]. Both French studies extracted data from the same source, the French National Discharge database. A nationwide retrospective study in Germany compared the care for acute ischemic stroke between the pandemic waves of 2019 and 2020. It provided gender-disaggregated data, revealing that the observed decline in overall stroke hospitalizations, especially during the second wave of the pandemic, was statistically more pronounced in women than in men. This difference could be attributed to behavioral factors, such as greater compliance with COVID-19 protective measures and a heightened perception of risk among women [20].

Among multi-national studies, the Global COVID-19 Registry, a retrospective multi-center cohort study that was conducted between March 2020 and June 2021 isolated 853 stroke/COVID cases from a total of 15,128 stroke patients from 105 centers worldwide. The investigators demonstrated a statistically significant male predominance, as male patients represented 57.9% of stroke/COVID cases but only 51% of non-COVID strokes (p<0.001). Of note, this registry included only stroke patients who received endovascular treatment and/or intravenous thrombolysis [24]. Similar results were derived from another multi-national study that explored the relationship between COVID-19 status and outcomes among patients with acute large vessel occlusion undergoing thrombectomy. In this cohort of 697 patients, 302 had COVID-19. The study compared stroke/COVID cases (302) to non-COVID strokes (395) recorded in stroke centers in North America, Europe, and the Middle East [25]. In this study, the percentage of female patients was 80.5% in the non-COVID cohort, but was found to be significantly lower, 41.1% (p<0.001), among stroke/COVID patients, denoting a male predominance in the latter group. The limitation in this study was that it included only stroke patients with confirmed large vessel occlusion who needed and received revascularization treatment.

Gender difference in outcomes

Stroke in COVID-19 patients appears to be associated with a greater mortality risk than stroke without COVID-19 [17,24], but it is not clear whether a gender difference in outcomes exists. Significantly fewer studies have tackled this question, and no meta-analyses are available. Two U.S. studies [14,18] had conflicting results, with Qureshi et al. reporting a worse disease outcome in men [18], but Katz et al. finding no difference in in-hospital mortality [14]. In the only study that specifically tackled the gender gradient in stroke outcomes, Trifan et al. conducted a retrospective study involving 83 acute stroke patients with COVID-19. The findings revealed that male patients with COVID-19 and stroke exhibited poorer survival and were less frequently discharged home than female ones; furthermore, the degree of disability, as expressed by the modified Rankin Stroke scale, was found to be higher in male patients, even after adjusting for confounders, such as the number of vascular risk factors present [15].

Gender differences in applied therapies

Based on relevant evidence [23-25], recanalization techniques in patients with stroke and COVID-19 appear to a) be less frequently used compared to non-COVID-19 stroke patients, despite the data that show their safety in this setting; b) exhibit longer door-to-intervention times; and, c) result in a poorer functional outcome and higher mortality. When it comes to gender-disaggregated data, an international controlled study focusing on MT in patients with stroke/COVID demonstrated that female sex was an independent predictor of favorable revascularization, as expressed by the rates of achievement of a grade 3 modified Thrombolysis in Cerebral Infarction [25]. Data from the Global COVID-19 stroke registry comprising 853 stroke/COVID cases from a total of 15,128 strokes showed that apart from the male predominance among stroke/COVID patients mentioned before, male patients more frequently underwent endovascular therapies (58% among the endovascular-therapies-only patients with COVID-19 vs. 48.5% among controls, p-value <0.001) [24]. Likewise, in the aforementioned French study by Gabet et al., men underwent MT more frequently than women (66% vs. 48%, respectively, p=0.02) [22]. Finally, in a European multi-center cohort study conducted between March 1, 2020, and May 5, 2020, MT registry of 93 stroke/COVID patients (out of 855 total stroke patients who received MT) showed a higher representation of males in this cohort (67.7%), without, however, including any form of comparison to contemporary or historical controls [23].

Discussion

This review highlights the lack of dedicated, gender-disaggregated data in the field of stroke as part of the neurological sequelae of COVID-19, providing a basis for additional investigation.

This paucity of data is mostly evident in regards to the outcomes and the applied therapies of stroke/COVID patients. Available data, albeit limited, point towards the direction of worse outcomes among men despite a more frequent use of advanced therapies in men compared to women. The latter is in accordance with other evidence of (i) gender discrimination in regards to the triage upon ICU admission (women are less frequently admitted to the ICU, as compared to men, despite more grave disease [26]), and (ii) a gender-driven differential in the treatment modalities offered to ICU patients (women have been found to receive less vital organ support within the ICU setting [27]). It is essential to emphasize that while our study does not specifically investigate gender discrimination in the ICU context, we contribute to the broader discussion on gender-related disparities in critical care. The existing literature highlights these disparities, and our findings underscore the need for further exploration in this area. From a pathophysiological point of view, it has been proposed that gender-specific differences in innate and adaptive immune responses, a higher concentration of inflammation markers and a more severe clinical course of COVID-19 in men, mainly due to slower control/resolution of infection and a more frequent transition to a prolonged, cytokine-driven, inflammatory state, might explain this sex differential among stroke/COVID patients [7,15].

However, when it comes to the question of the existence of a gender-driven gradient in the frequency of stroke occurrence, more data are available, albeit not gathered across studies in a pre-specified manner and with an aim to elucidate gender-driven differences. These data do not lead to a unanimous conclusion. In particular, studies from single countries, such as those from the USA [14,17-19] and one from France [22], exhibited no gender difference in the frequency of stroke/COVID. It appears intriguing to explain how another French study [21], with enrollment during a similar time period and recruitment of cases from the same electronic national database, yielded different results (i.e., a male predominance). Both French studies compared stroke/COVID patients to contemporary stroke patients without COVID-19. However, the study by Mariet et al. [21] confined data analysis regarding the gender gradient to the two lockdown months (March-May 2020), whereas the study by Gabet et al. [22] extended it to the whole study period, that is, January to mid-June 2020. It means that the latter study, apart from a longer enrollment period, included cases from January and February 2020, when COVID-19 cases were scarce. Another difference would be that the study by Gabet et al. [22] also included a subgroup (157 out of 800 stroke/COVID cases) of patients with a median delay of 19 days between the COVID-19 and the stroke hospitalizations, rendering a causal link doubtful.

In the case of registries [24,25], it is interesting to note that among stroke/COVID patients who received revascularization treatment, a statistically significant male predominance was found in contrast to the rather neutral studies mentioned above that included stroke/COVID cases irrespective of treatment. Since stroke/COVID appears to have a poorer prognosis among men, it would only be logical to assume that its entire clinical course would graver. This would lead to the observed over-representation of men among patients with confirmed large vessel occlusion [25] and among patients treated with advanced, more drastic, techniques, such as endovascular treatment [24].

Furthermore, significant meta-analyses have been conducted in the field of cerebrovascular events in patients with COVID-19. Nannoni et al. included 61 articles from around the globe in their meta-analysis, four of which accumulatively showed a non-significant OR of 0.92 (95% CI: 0.62-1.35) for women versus men to develop stroke in a total sample of 113 stroke/COVID cases out of 11,683 COVID-19 patients. However, when comparing stroke/COVID patients to non-COVID strokes (11 studies), women were significantly less frequently affected (OR=0.71; 95% CI: 0.51-0.99) [11]. A second meta-analysis that included a significant number of stroke/COVID cases (899) from 30 studies, mainly from the USA and the UK, reported a staggering male predominance of 70% of stroke/COVID cases, without, however, comparing to controls, either COVID-19 patients with no stroke, or stroke patients without COVID-19 [12]. Finally, a third meta-analysis, which extended data collection up to March 2021, found, among eight studies and 148 cases, the pooled proportions of female patients among stroke/COVID cases to be 36%, denoting a significant male predominance in frequency of occurrence (95%CI: 21-50%; p < 0.01) [13]. However, apart from the fact that the total number of included cases was rather small, one of the included studies, stemming from a single center in Abu Dhabi, the United Arab Emirates, displayed a marked male/female ratio of 18/1 [28], which surely influenced the pooled results. It is important to acknowledge some limitations of these meta-analyses. The above-mentioned meta-analyses were published rather early in the course of the pandemic and reflected an effort of the researchers to produce much needed statistically sound results from studies with a very limited number of stroke/COVID cases. Therefore, almost none of the studies presented herein had been included in these early meta-analyses. Moreover, the total number of included stroke/COVID patients in these meta-analyses is rather small, particularly when the meta-analysis of Siow et al. is excluded due to lack of control groups [12]. Conversely, certain single-national studies like the one by Gabet et al. were significantly larger [22] and therefore more statistically powered to detect gender-driven differences, although they were not specifically designed to do so.

Limitations

Our attempt to detect gender differences in stroke/COVID is hindered by certain limitations. Opting for a brief review, as opposed to a systematic or extensive one, aligns with our primary objective: delivering a succinct, focused, and accessible overview of the existing literature on stroke in COVID-19 patients.

While a meta-analysis based on selected studies could be valuable, our specific aim was to provide a quick and informative snapshot of the current research state. Extensive reviews typically involve a more thorough analysis, providing a detailed evaluation and discussion of the content, aiming to offer a nuanced assessment that aids readers in making well-informed decisions or gaining a deeper understanding of the subject matter. While we acknowledge the significance of meta-analyses and extensive reviews in certain contexts, we believe our concise format better serves our communication goals.

Moreover, the heterogeneity in study designs, patient populations, and data limitations rendered a meta-analysis unattainable. Practical considerations, including resource availability and the rapidly evolving nature of the COVID-19 pandemic, further influenced our choice. Opting for a brief review allowed us to disseminate relevant information promptly, synthesizing and presenting the most recent findings and insights efficiently. The constraints of limited time, human resources, and funding supported our decision to perform a focused review, enabling us to map existing literature, provide preliminary data on research questions, and offer a concise overview of the topic.

Since this was a brief review, the methodology of a systematic review, e.g., the PRISMA guidelines, was not followed. However, the steps that we followed are described in detail, as well as the way in which data were extracted. Overall, this brief review provides data and motivation for further research.

The very definition of “stroke in patients with COVID-19” has not been uniform in relevant studies: stroke patients with only a prior history of COVID-19 and not current COVID-19 have been included in studies, whereas other researchers have separated patients into those with COVID-19 as the primary diagnosis and those with COVID-19 as a merely accompanying, secondary diagnosis [19]; in other words, the concurrence of stroke with COVID-19 does not necessarily mean causality, especially if COVID-19 symptomatology is absent and/or lung imaging is unremarkable. This creates a non-uniform group of stroke patients in regards to their timing of exposure to COVID-19 and their clinical expression of SARS-CoV-2 infection, which, in turn, influences reported demographics and measured clinical outcomes.

Moreover, adjustment for confounders has been achieved with varying degrees of success among relevant studies, many of which relied heavily on propensity-score matching, as part of their retrospective design.

Another limitation would be that the majority of studies presented herein enrolled hospitalized patients; thus, any gender differences found cannot be generalized to outpatients diagnosed with COVID-19 who suffered mildly symptomatic strokes and were not transferred to healthcare facilities for various socioeconomic reasons or reasons related to restrictions due to COVID-19.

Lastly, relevant studies were clustered around the first year of the pandemic; there is a characteristic paucity of data concerning the later waves, when societies have abolished lockdowns, newer strains of the virus are reigning, and populations have been vaccinated against SARS-CoV-2 or have acquired immunity through prior infection. Follow-up studies on the cerebrovascular sequelae of COVID-19, with a focus on gender differences or inequalities, are warranted for policy-makers to fine-tune the responses of healthcare systems.

## Conclusions

All in all, when it comes to the gender-specific frequency of stroke in patients with COVID-19, the weight of the evidence appears to be against the presence of a significant difference in the representation of genders within the cohorts of stroke/COVID patients, although a tendency towards more frequent male representation cannot be excluded. In terms of management and outcomes, a worse clinical outcome in men against women is evident, along with an increased use of more advanced, endovascular therapies in male patients, probably due to a graver presentation and a less favorable clinical course.

Studies specifically designed and targeted to elucidate gender differences in stroke patients suffering from COVID-19 could have significant implications for clinical practice in critical care. If differences in outcomes, risk factors, or responses to treatment are indeed confirmed between male and female patients, it could lead to the development of enhanced prognostication and tailored treatment protocols and strategies. In that way, critical care personnel could create more personalized care plans that can account for potential variations in clinical presentation, response to treatment, and post-stroke rehabilitation needs; readily identify individuals who are at a higher risk of adverse outcomes, based on their gender; empower patients to understand their risks and contribute to their own recovery; and augment adherence to treatment plans and post-ICU care recommendations, including rehabilitation strategies and preventive measures against future strokes. Especially in the post-COVID era, healthcare systems are likely to be more attuned to the importance of gender-specific factors in patient care. In this light, incorporating the insights of the study of gender differences in the incidence, management, and outcomes of stroke in patients suffering from COVID-19 into everyday clinical practice is warranted, as it could result in more effective and patient-centered care for these patients, ultimately leading to improved outcomes, better quality of life, and better overall public health.
